# Predictive validity of early and mid-year literacy assessments for end-of-year word reading fluency

**DOI:** 10.1371/journal.pone.0327242

**Published:** 2025-06-25

**Authors:** Saeed Saad Alqahtani

**Affiliations:** Department of Special Education, College of Education, Prince Sattam bin Abdulaziz University, Al-Kharj, Saudi Arabia; Education University of Hong Kong, HONG KONG

## Abstract

This study explores the predictive validity of early and mid-year literacy assessments—Letter Naming Fluency (LNF), Letter Sound Fluency (LSF), and Phoneme Segmentation Fluency (PSF)—for end-of-year Word Reading Fluency (WRF) in kindergarten and grade 1 Arabic-speaking students. Using a longitudinal design, 412 students were assessed at three intervals (fall, mid-year, and year-end) to examine how these foundational literacy skills contribute to predicting WRF and identifying at-risk readers. Results from multiple regression analyses indicate that both LNF and LSF are significant predictors of end-of-year WRF across both fall and mid-year assessments, with mid-year measures demonstrating enhanced predictive accuracy. In contrast, PSF showed limited predictive value in comparison to LNF and LSF. Accuracy analyses revealed that mid-year assessments, particularly LNF and LSF, were more reliable in identifying at-risk students compared to fall assessments. Mid-year assessments correctly identified 64–73% of at-risk readers, emphasizing their value in early detection and intervention. These findings underline the importance of dynamic, multi-point assessments in improving educational practices and outcomes, particularly for students at risk of reading difficulties. The study highlights the critical role of foundational literacy skills in early reading development and provides actionable insights to enhance intervention strategies during key stages of literacy acquisition.

## Introduction

Learning to read involves developing skills to understand written language. It’s a process where language skills and code-related skills work together to help students read words and understand their meaning [1]. Thus, one of the first challenges for young learners is connecting symbols to the sounds to read words accurately and quickly [2]. To decode accurately at this stage, students must first acquire foundational literacy skills, including phonemic awareness and alphabetic knowledge [3].

Early literacy skills, as identified by the National Early Literacy Panel, are essential for reading development even before the acquisition of formal reading skills among English-language learners in the United States [4].These skills encompass letter-sound relationships and phonemic awareness [5,6]. Early literacy skills in the early grades can predict students’ decoding ability in following years [7,8] and overall reading success [5], underscoring the importance of nurturing these skills during the early grades.

### Predictive power of foundational literacy skills

Alphabetic knowledge and phonemic awareness are key predictors of reading achievements [9–11]. Alphabetic knowledge refers to the capacity to recognize printed letters and understand their associations with letter names and sounds. It comprises two main components: letter name knowledge, which indicate a student’s ability to match printed letters with their corresponding names, and letter sound knowledge, which indicate a student’s ability to match printed letters with their respective sounds [12,13]. Both skills are assessed using fluency-based measures which require students to name or pronounce letters within limited time (e.g., one minute) [14,15]. Alphabetic knowledge in early grades have strong relationship with reading achievement [16–18]. Thus, students who are at risk or with reading disabilities will have difficulties learn letter name and sounds [19,20].

Phonemic awareness refers to the ability to identify and manipulate individual sounds, or phonemes, in spoken language [3]. It involves recognizing that words are made up of separate sounds and being able to manipulate these sounds through activities like blending, segmenting, and manipulating phonemes in spoken words. One of the most common measures for phonemic awareness is Phoneme segmentation which require students to identify letter sounds within words. Phonemic awareness is essential for developing strong reading and spelling skills [21,22]. It is considered as the best predictor of reading skills [23–25]. Similar to alphabetic knowledge, students who are at risk or with reading disabilities have difficulties with phonemic awareness [25,26].

Letter naming fluency, letter sound fluency, and phoneme segmentation fluency are widely recognized as reliable predictors of word reading fluency (WRF). These measures provide crucial insights into students’ reading readiness, particularly for those at risk for reading difficulties. The speed and accuracy with which students perform these tasks correlate strongly with their ability to decode and recognize words fluently [16,18]. Consequently, these predictors are integral to identifying students who may require targeted interventions to achieve reading proficiency.

### Word reading fluency as a key literacy outcome

After mastering foundational literacy skills, students will develop the ability to decode and read words sufficiently. This proficiency serves as a prerequisite for progressing to more complex reading competencies, including vocabulary acquisition, fluency development, and comprehension strategies [27–29]. Word reading refers to the ability to accurately decode and recognize individual words in isolation, without the context of a larger text [30,31]. Proficient word reading requires students to read words with age- and grade-appropriate speed and accuracy [32,33]. The accepted levels of speed and accuracy vary depending on the student’s developmental stage and grade level expectations [34,35]. Within Curriculum-based Measurement (CBM) farmwork, word reading proficiency can be assessed requiring students to read a list of words in one minute [36,37]. Word reading skill can predict reading abilities and reading comprehension [38,39].

### Significance of the study

Prior research has emphasized the predictive value of early assessments such as Letter Naming Fluency (LNF), Letter Sound Fluency (LSF), and Phoneme Segmentation Fluency (PSF) for end-of-year outcomes [40]. However, much of this work has centered on single timepoints, such as fall or year-end assessments, overlooking the potential of mid-year data to enhance early identification and intervention.

The current study investigates the evolving predictive accuracy of LNF, LSF, and PSF between fall and mid-year assessments, demonstrating their ability to predict end-of-year Word Reading Fluency (WRF). Research supports the utility of mid-year assessments in identifying at-risk students earlier in the academic year [41,42]. This approach provides educators with the opportunity to implement timely, targeted interventions during the school year, an improvement over practices that rely solely on end-of-year data, which often leave limited time for effective remediation.

Although several studies have explored the development and validation of Arabic reading assessments [43–45], research in this area remains limited in scope and methodology. Prior studies have primarily focused on specific CBM tools such as Letter Naming Fluency (LNF) and Letter Sound Fluency (LSF) [46], word reading fluency [47,48], oral reading fluency [49], and reading comprehension using Maze tasks [50]. However, many of these studies are characterized by small sample sizes and limited technical adequacy, particularly in their ability to distinguish between skilled readers and students with reading difficulties. For example, Al-Hmouz [46] examined LNF and LSF performance in a sample of 150 students, while Abu-Hamour [48] evaluated word identification fluency in 75 students, divided between skilled readers and those with reading difficulties. Despite offering valuable benchmarks for instruction, these studies often faced methodological and analytical challenges that may limit the generalizability and practical application of their findings. Moreover, few studies have comprehensively assessed multiple components of early literacy, such as phonemic awareness, which are critical for the accurate identification of reading difficulties. This study addresses a significant gap in research by emphasizing how sequential assessments can be used to refine predictions over time. While studies have shown that LNF and LSF are robust predictors of WRF [42,51], few have systematically evaluated how predictive accuracy evolves from fall to mid-year. Additionally, while PSF is widely acknowledged for its role in phonological awareness and early reading skills [25], limited research has explored its specific predictive value at mid-year.

By addressing these gaps, this research provides both theoretical and practical contributions. The findings highlight the potential of mid-year assessments not only to enhance predictive accuracy but also to support real-time educational decision-making, offering insights into how assessment practices can be aligned with the dynamic nature of literacy development [52]. This perspective is supported by studies demonstrating that predictive models that incorporate multiple assessment periods outperform static models [53].

### Research questions

1. How do fall and mid-year assessments Letter Naming Fluency (LNF), Letter Sound Fluency (LSF), and Phoneme Segmentation Fluency (PSF) compare in their ability to predict end-of-year Word Reading Fluency (WRF) for kindergarten and grade 1 students?
2. What is the unique contribution of Letter Naming Fluency (LNF), Letter Sound Fluency (LSF), and Phoneme Segmentation Fluency (PSF) to the prediction of end-of-year word reading fluency (WRF) across fall and mid-year assessments?
3. To what extent do fall and mid-year assessments of Letter Naming Fluency (LNF), Letter Sound Fluency (LSF), and Phoneme Segmentation Fluency (PSF)accurately identify students at risk for reading failure by the end of the academic year?

## Methodology

### Participants

The study encompassed a sample of 412 Arabic-speaking students from kindergarten and first grade, enrolled during the academic year 2024. These participants were drawn from nine schools located in the central province of Saudi Arabia. The demographic composition of the sample was broadly representative of the Middle Eastern population, with no specific ethnic or social groups deliberately excluded. The gender distribution was balanced, with equal participation of males and females (50% each). Furthermore, the sample maintained an equal distribution across educational levels, with 50% of participants in kindergarten and 50% in first grade. The selection criteria involved choosing two classrooms from each participating school—one from kindergarten and one from first grade—resulting in a total of 18 classrooms being included in the study.

### CBM materials

Letter Naming Fluency (LNF): The researcher developed three distinct versions of test materials. Each version consisted of an A4 sheet displaying 28 Arabic letters arranged randomly across 10 rows and 15 columns, with some letters appearing more than five times throughout the sheet. Participants were instructed to read the letter names aloud, earning a point for each correctly identified letter within a one-minute time limit. The average correlation coefficient between the three versions was (.96).

Letter Sound Fluency (LSF): The LSF test followed a similar procedure to the LNF test, with the exception that the sheets presented letter sounds rather than letter names. Participants were asked to articulate both the letter sounds and names, receiving a point for each accurately identified sound within one minute. The average correlation coefficient between the versions was (.94).

Phoneme Segmentation Fluency (PSF): This assessment required participants to isolate the phonemes of words presented orally. The researchers created three versions of test materials, each containing 30 Arabic words and 96 syllables, aligning with the EasyCBM [54] criteria. The words were randomly distributed across the sheets prepared. Participants earned a point for each correctly identified phoneme. The average correlation coefficient between the versions was (.92).

Word Reading Fluency (WRF): Three versions of test materials were developed, each featuring an A4 sheet with 76 Arabic words arranged across 4 rows and 19 columns. Participants were tasked with reading the words aloud. The words were ranged in length from 2 letters to 6 but were randomly distributed across the sheet rather than being organized by number of letters level. Participants received a point for each correctly identified word. The average correlation coefficient between the versions was (.80).

The materials used in this study consisted of four assessment tools, each with three alternate forms, developed by the researcher based on well-established English-language CBM tools such as easyCBM, FAST, and DIBELS [54–56]. The Letter Naming Fluency (LNF) and Letter Sound Fluency (LSF) assessments consisted solely of Arabic letters. For the Phoneme Segmentation Fluency (PSF) and Word Reading Fluency (WRF) assessments, the word lists were developed using vocabulary drawn from the Saudi Arabian elementary Arabic school curriculum, ensuring that the most common and relevant words were represented. These tools have been used in previous research and were refined over time to improve their appropriateness and alignment with the target population. Additionally, the reliability of each test form was evaluated, and all forms demonstrated high internal consistency, with alternate form reliability coefficients exceeding 0.92.

The assessments were administered by two educators, both hold a bachelor’s degree in linguistics and had experience teaching language courses to elementary students. Prior to the study, the educators participated in a training session covering the research design and test implementation procedures. Over the course of one week, the researcher provided comprehensive explanations of the procedures, demonstrated the process, and allowed the educators to practice. Using a procedural checklist, the educators achieved an average accuracy of 95% when implementing the procedures. The research team conducted regular meetings to review the process and address any questions.

This study was reviewed and approved by the Standing Committee of Bioethics Research at Prince Sattam bin Abdulaziz University. The ethics approval number is [SCBR-195/2023], and the approval was granted on [12/19/2023]. After obtaining approval from the Institutional Review Board (IRB) and securing permission from the school district, the researcher contacted the school principals and obtained consent to conduct the study. Since the participants were young students, informed consent was obtained from their parents or legal guardians. Consent forms detailing the study’s purpose, procedures, and ethical considerations were sent to the parents, and written consent was secured prior to their children’s participation in the study. The assessments were administered individually to the participants. The same students were tested three times during the academic year (at the end of fall semester, at the end of winter semester, and at the end of spring semester). The recruitment of participants for this study took place between [1/1/2024] and [25/11/2024]. The study took place in the resource rooms of the selected schools. The educators provided participants with copies of the tests (except for the phonemic test) and utilized a stopwatch. The assessments were conducted daily over a two-week period at the end of each semester. In addition to the four tests, the educators completed a cover page for each participant, recording demographic information (e.g., age, grade, and sex). The educators were also instructed to record their voices during the test to facilitate reliability and procedural integrity procedures. The procedures and instructions were adapted from the CBM manual Book (Hosp et al., 2007). Furthermore, the classroom’s teachers assessed participants’ reading abilities based on their knowledge, assigning scores ranging from 1 to 10 each semester.

## Reliability and procedural integrity

To ensure the reliability of the scoring process, an independent educator, not involved in the scoring or rating, was assigned to review 10% of the randomly selected data. The educator listened to the recordings of each participant, evaluating both reliability and procedural integrity. Cronbach’s alpha values for the four tests demonstrated high reliability between the primary and secondary scorers, measuring.998,.992,.999, and.987 as an average. The educator utilized a checklist to assess procedural integrity, resulting in an average score of 95% (range, 92–100%). This indicated that the educators responsible for implementation diligently followed the assessment instructions.

### Analysis method

To answer the research questions, a series of analyses were conducted, including descriptive statistics, multiple regression analyses, hierarchical multiple regression analyses, and accuracy analysis for predicting at-risk students. The data were analyzed using JASP software [57].

#### 1. Descriptive statistics:

Descriptive statistics were calculated to summarize baseline data for each literacy measure (LNF, LSF, PSF) across the fall, winter, and spring semesters for kindergarten and grade 1. Means, standard deviations, minimum, and maximum scores were computed for each measure and are presented in Table 1.

**Table 1:** Descriptive Statistics.

Grades	Measures	Mean	SD	Min	Max
**Kindergarten**	LNF Fall	21.25	15.49	1.00	80.00
	LNF Winter	37.66	19.86	3.00	100.00
	LNF Spring	39.70	21.89	1.00	120.00
	LSF Fall	10.56	8.60	1.00	49.00
	LSF Winter	22.77	16.15	1.00	74.00
	LSF Spring	24.82	17.54	1.00	83.00
	WRF Fall	5.09	4.00	1.00	29.00
	WRF Winter	7.96	6.10	1.00	41.00
	WRF Spring	11.11	9.07	1.00	56.00
	PSF Fall	35.72	22.59	1.00	101.00
	PSF Winter	59.94	24.07	5.00	101.00
	PSF Spring	73.46	23.47	3.00	103.00
**Grade 1**	LNF Fall	49.63	19.96	1.00	120.00
	LNF Winter	65.41	21.84	7.00	110.00
	LNF Spring	68.37	22.34	18.0	128.00
	LSF Fall	30.67	17.55	1.00	71.00
	LSF Winter	40.66	19.73	5.00	89.00
	LSF Spring	43.94	20.03	5.00	95.00
	WRF Fall	14.57	9.75	2.00	47.00
	WRF Winter	19.83	12.40	3.00	62.00
	WRF Spring	23.13	13.48	3.00	76.00
	PSF Fall	60.63	17.82	12.00	101.00
	PSF Winter	81.72	17.16	15.00	101.00
	PSF Spring	90.80	16.17	36.00	103.00

#### 2. Multiple regression analysis:

To predict end-of-year word reading fluency (WRF), multiple regression analyses were conducted using each semester separately (fall and winter) scores of Letter Naming Fluency (LNF), Letter Sound Fluency (LSF), and Phoneme Segmentation Fluency (PSF) as predictors. Each model includes the three measures while word reading fluency by the end of year serve as dependent vibrable. Separate analyses were run for kindergarten and grade 1, allowing for an assessment of each measure’s unique contribution to the prediction of end-of-year WRF. Each predictor’s unstandardized coefficient (B), standard error (SE), standardized coefficient (β), t-value, and significance level (*p*) are provided in Table 2.

**Table 2:** Multiple Regression: Predicting end of year WRF status from fall and winter LNF, LSF, PSF for kindergarten and grade1.

Grade	Model	Outcomes vibrable included in the model	B	SE	*β*	t	*p*
**Kindergarten**	1	LNF1	0.19	0.060	0.33	3.20	.002*
		LSF1	0.40	0.109	0.38	3.70	.001*
		PSF1	−0.00	0.026	−0.01	−0.21	.830
	2	LNF2	0.224	0.040	0.492	5.581	.001*
		LSF2	0.167	0.049	0.303	3.427	.001*
		PSF2	0.035	0.022	0.094	1.622	0.107
**Grade 1**	1	LNF1	0.28	0.065	0.39	4.41	.001*
		LSF1	0.26	0.064	0.35	4.14	.001*
		PSF1	−0.04	0.051	−0.06	−0.85	.394
	2	LNF2	0.227	0.077	0.364	2.962	.004*
		LSF2	0.239	0.079	0.365	3.041	.003*
		PSF2	−0.002	0.066	−0.002	−0.023	.982

Note: LNF, letter naming fluency; LSF, letter sound fluency; PSF, phoneme segmentation fluency; B, unstandardized coefficient; SE, standard error; *β*, standardized beta; t, t-value; p, significance value; *p < 0.005”.

#### 3. Hierarchical multiple regression analysis:

To further investigate the incremental contributions of each literacy measure to WRF, hierarchical multiple regression was performed. Initial word reading ability (WRF1) was entered in the first block to control for prior reading skill, followed by LNF, LSF, and PSF in successive blocks to assess their unique contributions to end-of-year WRF. This method provided a clear picture of how each measure influenced end-of-year outcomes beyond prior ability. The hierarchical model results, including R^2^, F-values, p-values, and coefficients, are shown in Tables 3 and 4. These results clarify the impact of each predictor in explaining variance in end-of-year WRF.

**Table 3:** Hierarchical Multiple Regression Results: Predicting end of year WRF from fall LNF, LSF, PSF for kindergarten and grade1.

Grade	Model	Outcomes Vibrable included in the model	R^2^	*F*	*P*	B	β	*P*
**K**	1	WRF1	48.2	121.19	.001*	1.645	.695	.001*
	2	WRF1	51.2	67.70	.001*	1.213	.512	.001*
		LNF1				0.151	.251	.006*
	3	WRF1	53.6	49.27	.001*	1.052	.444	.001*
		LNF1				0.055	.091	.396
		LSF1				0.280	.264	.012*
	4	WRF1	53.7	36.76	.001*	1.054	.445	.001*
		LNF1				0.058	.097	.372
		LSF1				0.287	.271	.011*
		PSF1				−0.012	−.030	.660
**1**	1	WRF1	62.3	215.09	.001*	1.224	.789	.001*
	2	WRF1	62.9	109.45	.001*	1.115	.719	.001*
		LNF1				0.078	0.104	.153
	3	WRF1	62.9	72.40	.001*	1.114	0.718	.001*
		LNF1				0.077	0.104	.176
		LSF1				.00	0.001	.987
	4	WRF1	63.9	54.55	.001*	1.095	0.706	.001*
		LNF1				0.073	0.098	.206
		LSF1				0.005	0.007	.933
		PSF1				−0.040	−0.055	.319

Note: LNF, letter naming fluency; LSF, letter sound fluency; PSF, phoneme segmentation fluency; B, unstandardized coefficient; SE, standard error; *β*, standardized beta; t, t-value; p, significance value; *p < 0.005”.

**Table 4:** Hierarchical Multiple Regression Results: Predicting End-of-Year WRF from Mid-Year LNF, LSF, PSF for Kindergarten and Grade 1.

Grade	Model	Outcomes Vibrable included in the model	R^2^	F	p	*b*	β	*P*
**K**	1	WRF2	66.8	233.450	.001	1.158	0.817	.001*
	2	WRF2	71.8	146.623	.001	0.825	0.582	.001*
		LNF2				0.154	0.325	.001*
	3	WRF2	72.0	97.670	.001	0.788	0.556	.001*
		LNF2				0.138	0.290	.001*
		LSF2				0.038	0.069	.421
	4	WRF2	72.0	72.776	.001	0.778	0.549	.001*
		LNF2				0.137	0.288	.001*
		LSF2				0.037	0.068	.433
		PSF2				0.009	0.024	.667
**1**	1	WRF2	87.3	459.530	.001	1.096	0.934	.001*
	2	WRF2	87.8	236.439	.001	1.040,	0.886	.001*
		LNF2				0.056	0.084	.114
	3	WRF2	88.0	159.168	.001	1.074	0.915	.001*
		LNF2				0.086	0.130	.049
		LSF2				−0.056	−0.084	.234
	4	WRF2	88.9	127.525	.001	1.096	0.934	.001*
		LNF2				0.097	0.147	.024
		LSF2				−0.078	−0.116	.100
		PSF2				0.068	0.094	.032

#### 4. Accuracy analysis for identifying at-risk students:

An accuracy analysis was performed to assess each measure’s ability to identify students who fell below the 25th percentile in end-of-year WRF. Students were categorized based on their fall and spring LNF, LSF, and PSF scores, with accuracy rates calculated for each measure to determine predictive reliability. Table 5 summarizes the percentage accuracy for each measure in predicting end-of-year WRF performance. This analysis highlighted the efficacy of each measure, particularly emphasizing the predictive power of LNF and LSF in spring assessments over PSF.

**Table 5:** Accuracy in predicting students’ WRF performance.

Grade	Measure	Students who are below 25% percentile	Accuracy in predicting students’ performance in WR3 accurately
**k**	LNF1	21	45%
	LSF1	26	55%
	PSF1	20	43%
	LNF2	30	64%
	LSF2	28	60%
	PSF2	25	53%
**1**	LNF1	21	57%
	LSF1	22	59%
	PSF1	5	14%
	LNF2	25	68%
	LSF2	27	73%
	PSF2	14	38%

## Results

### Descriptive statistics

Table 1 presents the descriptive statistics for each grade (Kindergarten and First Grade) by measure and time point. The scores for each measure increased across the semesters (Fall, Winter, and Spring) for each grade. As expected, a large variation was observed across all measures.

### Multiple regression analysis

Table 2 presents the results of multiple regression analyses conducted to predict end-of-year word reading fluency (WRF) for kindergarten and first-grade students based on fall and winter assessment scores. The models were analyzed simultaneously for each grade to examine the contribution of early literacy predictors measured at different time points (fall and winter). The table includes the predictor variables included in each model, regression coefficients (B), standard error (SE), standardized coefficients (β), t-values, and p-values for each predictor.

To predict end-of-year word reading fluency (WRF) for kindergarten and grade 1 students, a multiple regression analysis was conducted using Letter Naming Fluency (LNF), Letter Sound Fluency (LSF), and Phoneme Segmentation Fluency (PSF) as predictors. The results are presented separately for each grade level.

For kindergarten, the regression model showed that LNF1 significantly predicted end-of-year WRF (β = 0.33, *p* = .002), and LSF1 also contributed significantly (β = 0.38, *p* = .001). However, PSF1 did not significantly contribute to the prediction of WRF (*p* = .830). For the second semester, LNF2 was the largest significant predictor (β = 0.492, *p* < .001), followed by LSF2 (β = 0.303, *p* < .001). PSF2 did not contribute significantly (*p* = .107).

For grade 1, LNF1 (β = 0.39, *p* < .001) and LSF1 (β = 0.35, *p* < .001) were similar significant predictors of WRF, while PSF1 was not significant (*p* = .394). In the second semester, LNF2 (β = 0.364, *p* = .004) and LSF2 (β = 0.365, *p* = .003) remained significant predictors, while PSF2 did not significantly contribute (*p* = .982).

In summary, for both grades, initial and mid-year letter naming fluency (LNF) and letter sound fluency (LSF) were consistently significant predictors of end-of-year word reading fluency. However, phoneme segmentation fluency (PSF), both at the start and mid-year, did not show significant predictive power in either grade, suggesting it may play a less prominent role in end-of-year word reading outcomes.

### Hieratical multiple regression analyses for fall assessments

Table 3 presents the results of hierarchical multiple regression analyses conducted to predict end-of-year word reading fluency (WRF) for kindergarten and first-grade students based on fall assessment scores. For each grade, four models were analyzed, with predictor variables added sequentially to examine their incremental contribution to predicting WRF. The table includes the predictor variables included in each model, the explained variance (R^2^) for each model, the results of the analysis of variance (ANOVA) test, and the regression coefficients for each predictor.

To predict end-of-year word reading fluency (WRF) for kindergarten and grade 1 students, a hierarchical multiple regression analysis was conducted with fall word reading (WRF1) entered in the first block to control for initial reading skill, followed by Letter Naming Fluency (LNF1), Letter Sound Fluency (LSF1), and Phoneme Segmentation Fluency (PSF1) in subsequent blocks. Results for each grade are as follows:

Initial WRF1 accounted for 48.2% of the variance in end-of-year WRF (R^2^ = 0.482, F = 121.19, p < .001), with a significant coefficient (β = .695, p < .001). Adding LNF1 increased the explained variance by 3.0% to a total of 51.2% (R^2^ = 0.512, F = 67.70, p < .001), with LNF1 significantly predicting WRF (β = .512, p = .006). Including LSF1 further increased the explained variance to 53.6% (R^2^ = 0.536, F = 49.27, p < .001), with a significant coefficient (β = .264, p = .012). However, adding PSF1 contributed only 0.1% additional variance, leaving the model at 53.7% explained variance (R^2^ = 0.537, F = 36.76, p < .001), and PSF1 did not significantly predict WRF (β = −.030, p = .660).

For grade 1, initial WRF1 accounted for 62.3% of the variance in end-of-year WRF (R^2^ = 0.623, F = 215.09, p < .001), with a significant coefficient (β = .789, p < .001). Adding LNF1 slightly increased the explained variance to 62.9% (R^2^ = 0.629, F = 109.45, p < .001), but LNF1 did not significantly predict WRF (β = .104, p = .168). Including LSF1 kept the explained variance at 62.9% (R^2^ = 0.629, F = 72.40, p < .001), with no significant contribution (β = .001, p = .987). Finally, adding PSF1 did not increase the explained variance further (R^2^ = 0.639, F = 54.55, p < .001), and PSF1 also showed no significant effect (β = −.055, p = .319).

In Summary, for kindergarten, WRF1, LNF1, and LSF1 were significant predictors of end-of-year WRF, with LNF1 and LSF1 each adding unique variance. PSF1, however, did not contribute significantly. For grade 1, only initial WRF1 was a significant predictor, while LNF1, LSF1, and PSF1 did not contribute additional significant variance in predicting end-of-year WRF. PSF1 did not.

### Hieratical multiple regression analyses for mid-year assessments

Table 4 presents the results of hierarchical multiple regression analyses conducted to predict end-of-year word reading fluency (WRF) for kindergarten and first-grade students based on winter assessment scores. For each grade, four models were analyzed, with predictor variables added sequentially to examine their incremental contribution to predicting WRF. The table includes the predictor variables included in each model, the explained variance (R^2^) for each model, the results of the analysis of variance (ANOVA) test, and the regression coefficients for each predictor.

For Kindergarten, initial WRF2 accounted for 66.8% of the variance in end-of-year WRF (R^2^ = 0.668, F = 233.450, p < .001), with a significant coefficient (β = 0.817, p < .001). Adding LNF2 increased the explained variance by 5% to a total of 71.8% (R^2^ = 0.718, F = 146.623, p < .001), with LNF2 significantly predicting WRF (β = 0.325, p < .001). Including LSF2 slightly increased the explained variance to 72.0% (R^2^ = 0.720, F = 97.670, p < .001), with LSF2 not contributing significantly (β = 0.069, p = .421). Adding PSF2 did not further improve the explained variance, leaving the model at 72.0% (R^2^ = 0.720, F = 72.776, p < .001). PSF2 did not significantly predict WRF (β = 0.024, p = .667).

For grade 1, initial WRF2 accounted for 87.3% of the variance in end-of-year WRF (R^2^ = 0.873, F = 459.530, p < .001), with a significant coefficient (β = 0.934, p < .001). Adding LNF2 slightly increased the explained variance to 87.8% (R^2^ = 0.878, F = 236.439, p < .001), though LNF2 was not a significant predictor (β = 0.084, p = .114). Including LSF2 raised the explained variance to 88.0% (R^2^ = 0.880, F = 159.168, p < .001), with LNF2 becoming significant (β = 0.130, p = .049) but LSF2 not contributing significantly (β = −0.084, p = .234). Adding PSF2 increased the explained variance to 88.9% (R^2^ = 0.889, F = 127.525, p < .001), with PSF2 showing a small but significant contribution (β = 0.094, p = .032).

In summary, for kindergarten, WRF2 and LNF2 were significant predictors of end-of-year WRF, with LNF2 adding unique variance to the model. LSF2 and PSF2, however, did not contribute significantly to the prediction. For grade 1, WRF2 remained the primary significant predictor of end-of-year WRF. While LNF2 added a small but significant contribution to the model at certain stages, LSF2 and PSF2 did not contribute additional significant variance in predicting end-of-year WRF.

### Accuracy in predicting at risk students for reading failure

The performance of students who scored below the 25th percentile in word reading fluency (WRF) by the end of the academic year was examined. These students were categorized, and their scores on Letter Naming Fluency (LNF), Letter Sound Fluency (LSF), and Phoneme Segmentation Fluency (PSF) from the fall and spring semesters were compared to assess the accuracy of these measures in predicting end-of-year WRF performance. The analysis aimed to determine the extent to which students who fell below the 25th percentile in WRF by the end of the academic year could be predicted based on their performance in the fall and spring assessments. A total of 47 kindergarten students and 37 grade 1 student were identified as falling below the 25th percentile in WRF by the end of the academic year. A test conducted between students who are below 25th percentile in WRF by the end of the academic year and the rest of the students. The results shows a significant differences between the groups for each grade (M = 3.19, for at risk students, M = 14.29, for non at risk *t* = −8.49, p < .001) for Kindergarten and (M = 9.02, for at risk students, M = 28.36, for non at risk, *t* = −9.64, p < .001).

For kindergarten students using fall measures, 45% of students were correctly identified as at-risk based on LNF1, while LSF1 showed a higher accuracy of 55% in predicting WRF performance by the year’s end. PSF1 was less predictive, with an accuracy of 43%. Spring measures showed improved accuracy, with 64% of students correctly classified using LNF2 and 60% with LSF2. PSF2 identified 53% accurately, indicating better predictive alignment in spring assessments.

For grade 1 students, Fall LNF1 and LSF1 scores identified 57% and 59% of students, respectively, as at-risk, with PSF1 showing a much lower accuracy of 14%. Spring measures proved more effective, with LNF2 achieving 68% accuracy, LSF2 73%, and PSF2 identifying 38% correctly. These results highlight that spring assessments, particularly LNF2 and LSF2, had higher predictive accuracy for both kindergarten and grade 1 students, while fall assessments showed moderate accuracy. PSF measures were generally less predictive, especially in the fall, indicating the greater reliability of letter fluency measures over phoneme segmentation in predicting end-of-year WRF performance.

## Discussion

This study aimed to investigate the predictive validity of fall and mid-year literacy assessments, specifically Letter Naming Fluency (LNF), Letter Sound Fluency (LSF), and Phoneme Segmentation Fluency (PSF), in predicting end-of-year Word Reading Fluency (WRF) among kindergarten and grade 1 Arabic-speaking students. These assessments serve various educational purposes, including screening, monitoring progress, and identifying children at risk for reading difficulties. The findings contribute to the existing body of research by highlighting the evolving predictive power of these measures over the academic year, with a particular emphasis on the utility of mid-year assessments.

### Predictive role of letter naming fluency and letter sound fluency

Letter naming fluency in fall and winter were significant predictors of WRF by the end of the year for both grades. However, using Hierarchical Multiple Regression, the unique strongest predictors for WRF was winter LNF for kindergarten, following by similar amount of predictions for fall and winter semesters range from.33 to 39 for both grades. In other words, assessing LNF for kindergarten and first grade in fall or winter can predict students’ words reading skills by the end of their academic year. LNF contribute to the prediction of later decoding acquisitions, this result has been replicated by many studies [4,18,40]. Learning letter naming helps young students move to the second stage in reading by mastering the ability to understand the words, and spelling especially for kindergarten children. Letter name knowledge enhances the connection between letters and sounds, which is fundamental for reading [58]. In the same time, improvements in LNF during the fall semester significantly predict better word reading outcomes later, suggesting that early gains in LNF are crucial. Thus, teaching letter names alongside letter sounds strengthens this relationship, highlighting its instructional value in early education [14].

Letter sound fluency requires students to pronounce the sounds of letters, which demands more in-depth decoding abilities than LNF. Consequently, the average scores for LSF were lower than those for LNF. LSF in fall and winter were significant predictors of WRF by the end of the year for both grades. However, using Hierarchical Multiple Regression, all LSF in fall and winter for both grades were uniquely strong predictors for WRF with similar amount of predictions. LSF contribute to the prediction of later decoding acquisitions, this results consistent with several studies [18,40,51,59]. Compared to LNF, students learn more advance step in learning letters sounds which requires to link the between sounds and letters that are shaping those sounds (i.e., grapheme–phoneme correspondences), which are the fundamental step to understand words and read them correctly. In the same time, improvements in LNF during the fall semester significantly predict better word reading outcomes later, suggesting that early gains in LSF are crucial. Thus, teaching letter sounds alongside letter sounds strengthens this relationship, highlighting its instructional value in early education. Teaching letter-sound fluency significantly improves reading fluency and word reading skills in children, including those with dyslexia [60,61].

### Limited predictive value of phoneme segmentation fluency

Although there was a moderate to weak correlation between PSF and other measures, PSF does not significantly contribute to the prediction of word reading compared to LNF or LSF. Phoneme segmentation fluency (PSF) was not significant predictors of WRF for any grade in any times. In other studies, phonemic segmentation is a strong predictor of reading and spelling abilities in children, outperforming other phonological skills such as rhyme and alliteration sound categorization [21,62–64]. However, [40] found a weaker effect of PSF on word reading developments compared to LSF. This is understandable because training in phoneme segmentation significantly improves reading readiness and early reading skills in kindergarten students (Ball & Blachman, 1988; Spector, 1992). One explanation is the time taking the test (intercept), the test administration in this study took place at the end the semester one, where other studies [40] administrate PSF at the beginning of the academic year before the students start learn. in their study, the PSF mean at the beginning at the end of fall increased from 7 at the beginning of the fall semester to 19 by the end of the same semester.

### Fall vs. mid-year assessments

Mid-year assessments demonstrated significantly greater predictive power for the end-of-year Word Reading Fluency (WRF) compared to fall assessments, particularly in grade 1. For kindergarten, mid-year assessments explained 72% of the variance in WRF, compared to 53.7% in the fall, reflecting a 34% improvement in predictive accuracy. This trend was even more pronounced in grade 1, where mid-year R^2^ values exceeded 87%, compared to 63.9% for fall assessments, marking a substantial 36% increase. These findings are consistent with prior research highlighting the cumulative benefits of literacy instruction, which provide students with greater exposure to foundational skills, such as grapheme-phoneme correspondences and fluency development, over the course of the academic year [14,40]. The enhanced predictive accuracy of mid-year assessments underscores their value in capturing the progression of literacy skills, particularly as students begin to integrate phonological and orthographic knowledge into fluent word recognition [58,59].

### Accuracy of fall and mid-year assessments in identifying at-risk students

The accuracy analyses demonstrated that mid-year assessments are consistently more reliable in identifying at-risk students compared to fall assessments. For kindergarten, mid-year LNF correctly identified 64% of students below the 25th percentile in WRF, a marked improvement over the 45% accuracy observed in the fall. Similarly, mid-year LSF outperformed its fall counterpart (60% vs. 55%), further supporting the assertion that additional literacy instruction enhances the predictive validity of mid-year assessments [41,43]. These findings are in line with studies indicating that LNF and LSF are strong indicators of reading risk, particularly when measured after students have received foundational literacy instruction [18,60]. In grade 1, the accuracy gains were even more pronounced, with mid-year LSF achieving the highest accuracy across all measures (73%), underscoring its importance for identifying at-risk readers at this stage of literacy development [19,61]. PSF, while the least accurate measure overall, improved from fall to mid-year in both grades, a finding consistent with research suggesting that phoneme segmentation skills develop more slowly and may have limited utility for predicting word reading fluency [40,62]. These results suggest that educators should prioritize mid-year assessments, particularly LNF and LSF, to maximize the effectiveness of literacy screenings and interventions during critical instructional periods.

### Implications

The results of this study offer several important implications for educational practice, particularly in the context of early literacy instruction for Arabic-speaking students. The findings emphasize the importance of using multiple assessment points throughout the academic year, rather than relying solely on initial screening at the beginning of the school year. Mid-year assessments of Letter Naming Fluency (LNF) and Letter Sound Fluency (LSF) were found to substantially improve the accuracy of identifying students at risk for reading difficulties. This has direct relevance for schools seeking to implement effective early intervention strategies.

First, these results support the use of dynamic, curriculum-based measurement (CBM) tools as part of a systematic progress-monitoring framework. Teachers can use mid-year CBM assessments to track students’ development in foundational literacy skills and to adjust instruction based on individual student needs. Regular assessment enables timely interventions before reading difficulties become more severe, aligning with best practices in early intervention. Second, the findings highlight the importance of data-driven instruction. Providing teachers with professional development on how to administer, interpret, and utilize CBM data can empower them to make informed instructional decisions. Such training ensures that teachers are not only using assessments for identification but also integrating results into instructional planning.

Third, school administrators and policymakers should consider embedding multi-point assessments within the curriculum and literacy policies. This may involve allocating resources for assessment materials, teacher training, and intervention programs designed to support students who are identified as at-risk based on mid-year performance. Lastly, the study contributes to the broader field of literacy education in Arabic, where empirical research on early assessment practices remains limited. Implementing evidence-based assessment practices tailored to the linguistic characteristics of Arabic can support more effective teaching practices and improve reading outcomes for students in the early grades.

### Limitations

Although the results are promising, several limitations should be taken into consideration. First, the study was conducted at the end of each semester. This timing was intentionally selected to represent actual real-life scenarios where teachers identify students with reading difficulties after working with them for an extended period. The goal of this approach was to capture real-world challenges and examine the issue from different perspectives. However, this method may limit the ability to capture ongoing learning progress throughout the semester. However, this was not the primary focus of the current study. Second, the study relied on a relatively short-term follow-up period, focusing on a single academic year, which restricts the ability to examine the long-term predictive power of these measures on reading outcomes. Third, although phoneme segmentation fluency (PSF) is widely used to measure phonological awareness, it may not fully capture the predictive validity of phonemic skills for end-of-year word reading fluency. Other phonemic measures, such as phoneme blending tasks, where students combine individual phonemes to form words, or elision tasks, which require students to manipulate phonemes by adding or omitting sounds, have been shown to provide stronger predictive power for reading outcomes [18,65].
